# A Newly Uncovered Group of Distantly Related Lysine Methyltransferases Preferentially Interact with Molecular Chaperones to Regulate Their Activity

**DOI:** 10.1371/journal.pgen.1003210

**Published:** 2013-01-17

**Authors:** Philippe Cloutier, Mathieu Lavallée-Adam, Denis Faubert, Mathieu Blanchette, Benoit Coulombe

**Affiliations:** 1 Institut de Recherches Cliniques de Montréal (IRCM), Montréal, Québec, Canada; 2 McGill Centre for Bioinformatics and School of Computer Science, McGill University, Montréal, Québec, Canada; 3 Department of Biochemistry, Université de Montréal, Montréal, Québec, Canada; Stanford University School of Medicine, United States of America

## Abstract

Methylation is a post-translational modification that can affect numerous features of proteins, notably cellular localization, turnover, activity, and molecular interactions. Recent genome-wide analyses have considerably extended the list of human genes encoding putative methyltransferases. Studies on protein methyltransferases have revealed that the regulatory function of methylation is not limited to epigenetics, with many non-histone substrates now being discovered. We present here our findings on a novel family of distantly related putative methyltransferases. Affinity purification coupled to mass spectrometry shows a marked preference for these proteins to associate with various chaperones. Based on the spectral data, we were able to identify methylation sites in substrates, notably trimethylation of K135 of KIN/Kin17, K561 of HSPA8/Hsc70 as well as corresponding lysine residues in other Hsp70 isoforms, and K315 of VCP/p97. All modification sites were subsequently confirmed in vitro. In the case of VCP, methylation by METTL21D was stimulated by the addition of the UBX cofactor ASPSCR1, which we show directly interacts with the methyltransferase. This stimulatory effect was lost when we used VCP mutants (R155H, R159G, and R191Q) known to cause Inclusion Body Myopathy with Paget's disease of bone and Fronto-temporal Dementia (IBMPFD) and/or familial Amyotrophic Lateral Sclerosis (ALS). Lysine 315 falls in proximity to the Walker B motif of VCP's first ATPase/D1 domain. Our results indicate that methylation of this site negatively impacts its ATPase activity. Overall, this report uncovers a new role for protein methylation as a regulatory pathway for molecular chaperones and defines a novel regulatory mechanism for the chaperone VCP, whose deregulation is causative of degenerative neuromuscular diseases.

## Author Summary

Methylation, or transfer of a single or multiple methyl groups (CH_3_), is one of many post-translational modifications that occur on proteins. Such modifications can, in turn, affect numerous aspects of a protein, notably cellular localization, turnover, activity, and molecular interactions. In addition to post-translational modifications, the structural organization of a protein or protein complex can also have a significant impact on its function and stability. A group of factors known as “molecular chaperones” aid newly synthesized proteins in reaching their native conformation or alternating between physiologically relevant states. We present here a new family of factors that promote methylation of chaperones and show that, at least in one case, this modification translates into a modulation in the activity of the substrate chaperone. Our results not only characterize the function of previously unknown gene products, uncover a new role for protein methylation as a regulatory pathway for chaperones, and define a novel regulatory mechanism for the chaperone VCP, whose deregulation is causative of neuromuscular diseases, but also suggest the existence of a post-translational modification code that regulates molecular chaperones. Further decrypting this “chaperone code” will help understanding how the functional organization of the proteome is orchestrated.

## Introduction

Methyltransferases catalyze the transfer of a methyl group (CH_3_) from a donor, generally S-adenosyl-L-methionine (AdoMet), to various acceptor molecules such as proteins, DNA, RNA, lipids, and small metabolites [1]–[3]. Protein N-methylation predominantly targets the side chains of two amino acids, lysine and arginine, whereas the side chains of other residues, including histidine, glutamine, and asparagine represent minor targets for methylation [4]–[6]. Dicarboxylic amino acids (glutamate, aspartate) and cysteine are also known to be respectively O- and S-methylated on occasion [7], [8]. In addition, some proteins were shown to be methylated on their N-terminal and C-terminal ends [9]–[11]. The vast majority of methyltransferases are grouped into three large families based on their structure, namely seven-β-strand, SET and SPOUT domain-containing methyltransferases [2]. All protein R methyltransferases (PRMT) are part of the seven-β-strand superfamily, while protein K methyltransferases (PKMT) fall almost exclusively within the SET domain-containing group. Until recently, the only known seven-β-strand PKMT was Dot1 [12].

Efforts to characterize substrates of PKMT have mostly focused on nucleosome components. Methylation of histone H3 residues K4, K36, and K79 are associated with transcriptionally active euchromatin, whereas methylation of H3K9, H3K27 and H4K20 coincides with heterochromatin and transcriptional repression [13], [14]. Recent reports have furthermore shown that the type of lysine methylation (i.e., mono-, di- or trimethylation) should also be taken into consideration when assessing chromatin state [15]–[17]. Epigenetics has been paramount in demonstrating that a modification as seemingly insignificant as the addition of a methyl group can have a considerable impact on a biological process as crucial as gene expression. Evidence of lysine methylation-driven regulation has been documented for an ever-increasing number of non-histone proteins, including calmodulin, cytochrome C, Rubisco, ribosomal proteins, p53, and NF-κB [18]–[27].

As part of an effort to systematically map protein-protein interactions, we came across a previously uncharacterized protein sharing distant homology with PRMTs nestled within a network of molecular chaperones involved in protein complex assembly. Subsequent local alignement searches using that protein as seed uncovered a group of 10 distantly related putative methyltransferases. Characterization of the interaction network of this novel subgroup of methyltransferases was undertaken by Affinity Purification coupled to Mass Spectrometry (AP-MS) and then computationally assessed. Our results revealed that enzymes of this subgroup preferentially interact with molecular chaperones. Validation experiments using three of the identified interactors, Kin17, Hsc70, and VCP/p97, indicated that they represent bona fide substrates. In each case, trimethylated lysine residues were identified in vivo and confirmed in vitro using recombinant methyltransferase-substrate pairs. In addition, we have shown that methylation of one of these substrates, VCP/p97, by METTL21D can be modulated by ASPSCR1/UBXD9 and that this modification regulates ATPase activity of the VCP chaperone. The results presented here bring to light an entirely new cast of PKMTs of the seven-β-strand variety and expands our knowledge of non-histones substrates, most notably molecular chaperones. This finding points to a new role for protein methylation in regulating protein folding, quality control, and turnover.

## Results

### Characterization of a newly discovered family of distantly related putative methyltransferases

**Figure 1:**
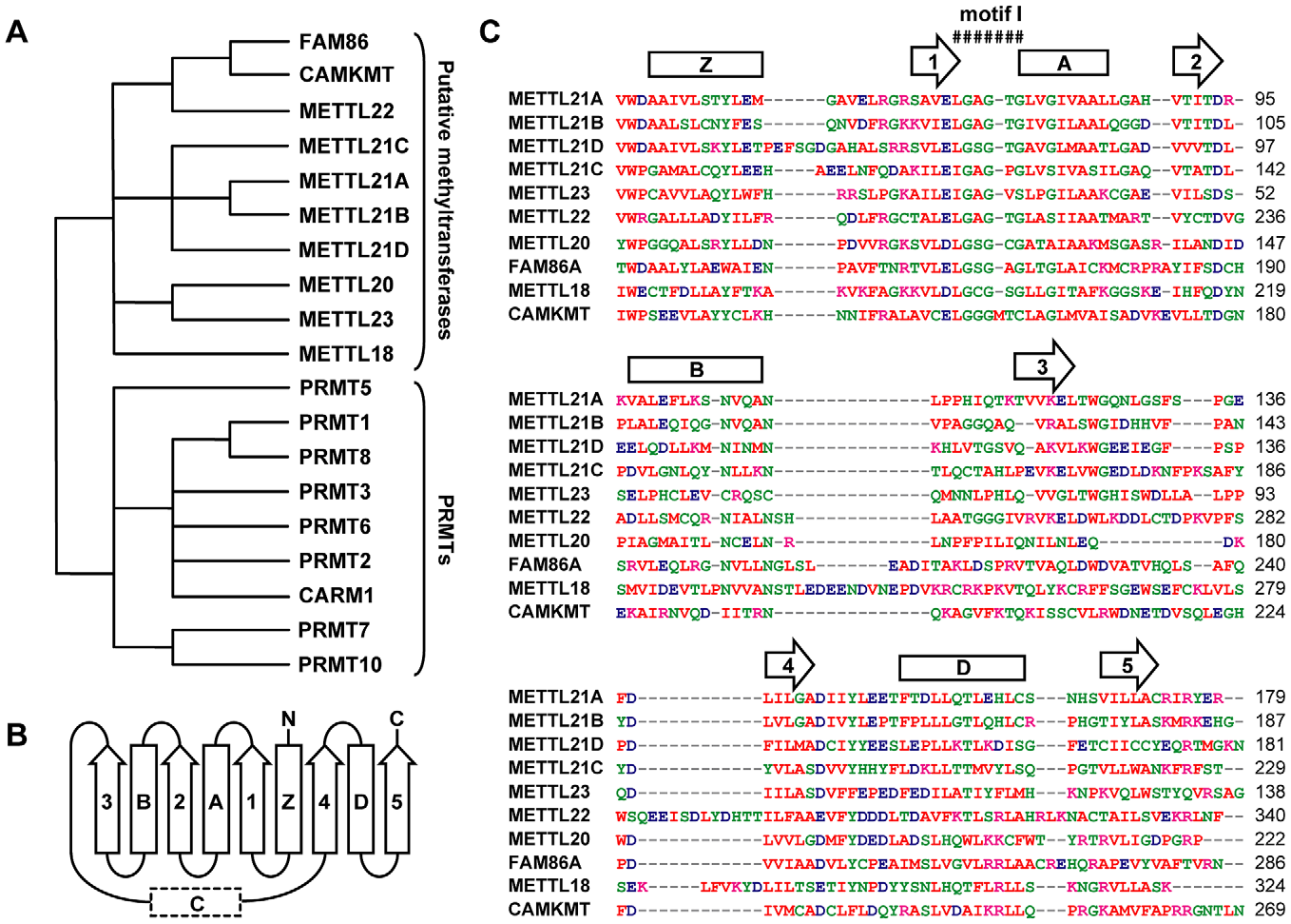
Computational analysis defines a novel family of putative protein methyltransferases. (A) Unrooted phylogenetic tree of a family of human putative methyltransferases distantly related to PRMTs. FAM86 represents a number of genetic variants (FAM86A, B1, B2, C, and D) whose duplication is observed only in primates. Branch lengths are not proportional to the actual evolutionary distances between the sequences. (B) Secondary structure organization of the Rossmann fold domain of PRMTs responsible for the methyltransferase activity. Arrows represent β strands and rectangles correspond to α helices, including typically ill-defined or inexistent α helix C. (C) Multiple sequence alignment of the Rossmann fold of all members within this family as generated by ClustalW2 (http://www.ebi.ac.uk/Tools/msa/clustalw2/). Red residues are small, hydrophobic, aromatic; blue are acidic; magenta are basic; and all other residues are green. Primary sequence alignment corresponds nicely with secondary structure prediction by Jpred3 (http://www.compbio.dundee.ac.uk/www-jpred/). Overhead β strands and α helices are shown as in (B). Conserved motif I, the site of S-adenosylmethionine binding, is also marked.

The study of this group of previously uncharacterized methyltransferases was initiated when METTL22 was identified in the soluble fraction of a protein affinity purification that targeted the DNA/RNA binding protein Kin17/KIN. Local alignment searches were performed to ascertain the function of this protein (data not shown). It was discovered that METTL22 was part of a larger group of 10 proteins (if the diversity of FAM86 closely-related isoforms are considered as a single member) that shared distant homology with PRMTs. Phylogenetic analysis of the most conserved region of these two protein groups (Figure S1) confirmed this observation, suggesting that this family of uncharacterized methyltransferases is related to, but distinct from, PRMTs (Figure 1A; Figure S2). Computational structure prediction further demonstrated the similarity between the members of this family of putative methyltransferases and PRMTs (Figure 1B, 1C). The subsequent publication of the human “methyltransferasome” by Clarke and colleagues confirmed that these proteins are putative methyltransferases and that they form a distinct family [2]. Indeed, most of the methyltransferases described here fall within the so-called “Group J.”

**Figure 2:**
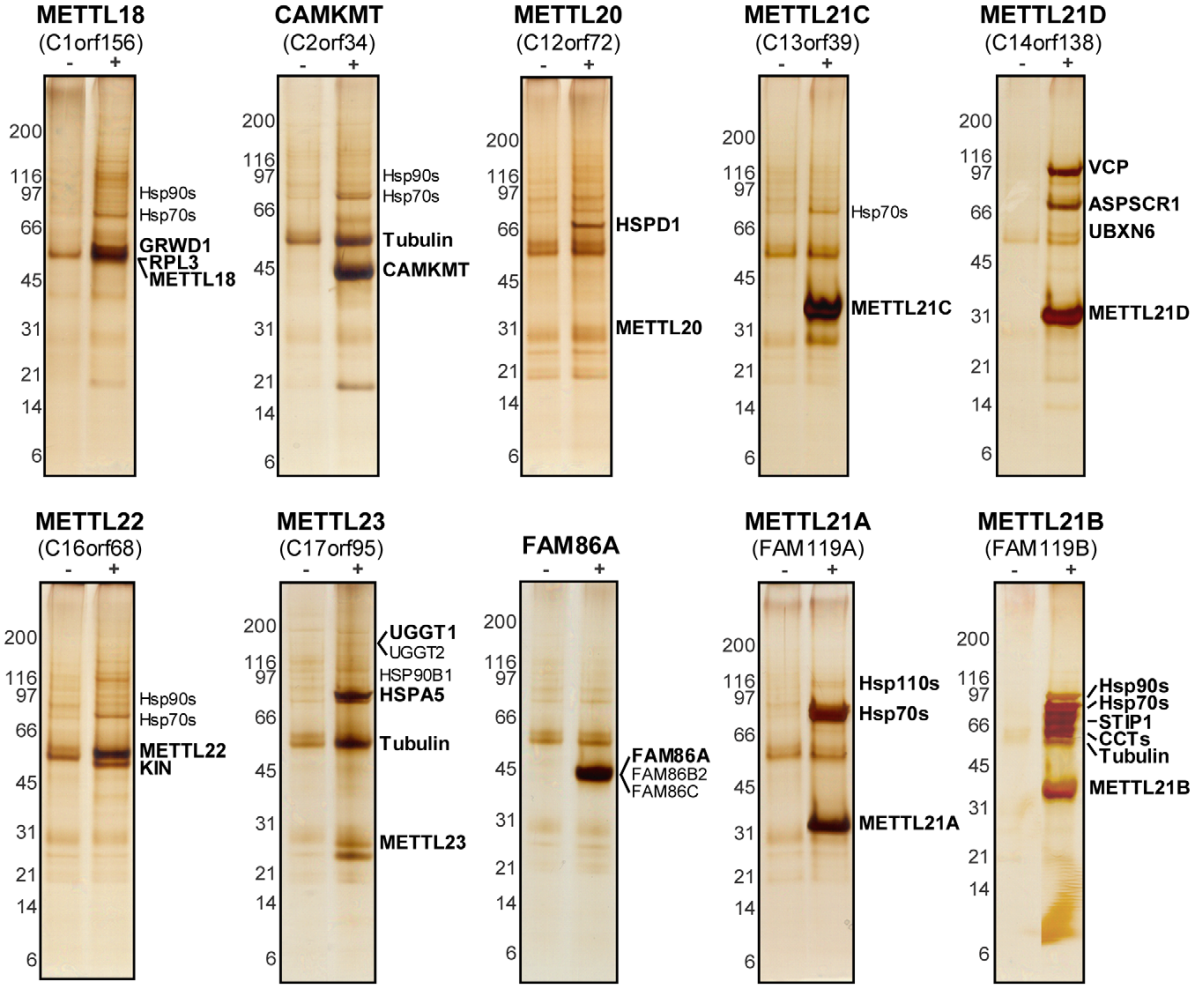
Tandem-affinity purification coupled to mass spectrometry reveals protein interaction network of putative methyltransferases. Purification of 10 TAP-tagged putative methyltransferases from ponasterone-inducible strains of HEK 293 cells. Eluted proteins were separated by SDS-PAGE. Gels were silver stained and cut in slices that were then trypsin digested before protein identification by LC-MS/MS. Tagged baits and major interactors are marked.

Based on the observed homology with PRMTs, we hypothesized that these proteins were likely protein methyltransferases themselves. To identify possible substrates and cofactors, we elected to map the protein interaction network for each member of this novel family by AP-MS (main interactors are marked in Figure 2, additional targets are listed in Table S1) [28]–[35]. The main METTL22 interactor identified in the soluble fraction was KIN, which confirmed our initial observation and further strengthened the notion that these two factors interact. A common theme for most of these putative methyltransferases' interactors was chaperones, be they of the Hsp70 or Hsp90 variety (see METTL18, CAMKMT, METTL21C, METTL22, METTL23, METTL21A, and METTL21B), chaperonins HSPD1 and CCT (see METTL18, CAMKMT, METTL20, and METTL21B), and even the AAA ATPase VCP (see METTL21D) that is believed to act as a chaperone in various processes, most notably Endoplasmic Reticulum-Associated Protein Degradation (ERAD) [36]–. Recent publications have substantiated the accuracy of this interactome. Firstly, it was demonstrated in another report by Steven Clarke on the yeast homolog of METTL18, YIL110W, that this protein methylates the ribosomal protein RPL3 [6]. Our own purification of METTL18 identified RPL3 and its associated ribosome biogenesis factor GRWD1 [41] as the two main interactors. Secondly, CAMKMT has been shown to methylate calmodulin on a lysine residue [42]. We were likewise able to co-purify calmodulin protein CALM2 in our CAMKMT affinity purification. However, it should be noted that since our AP-MS protocol includes beads bearing the calmodulin-binding peptide (CBP), calmodulin often appears as a non-specific target, although usually with a weaker signal. Computational assessment showed that CALM2 was a high confidence interactor of CAMKMT (FDR<10%).

**Figure 3:**
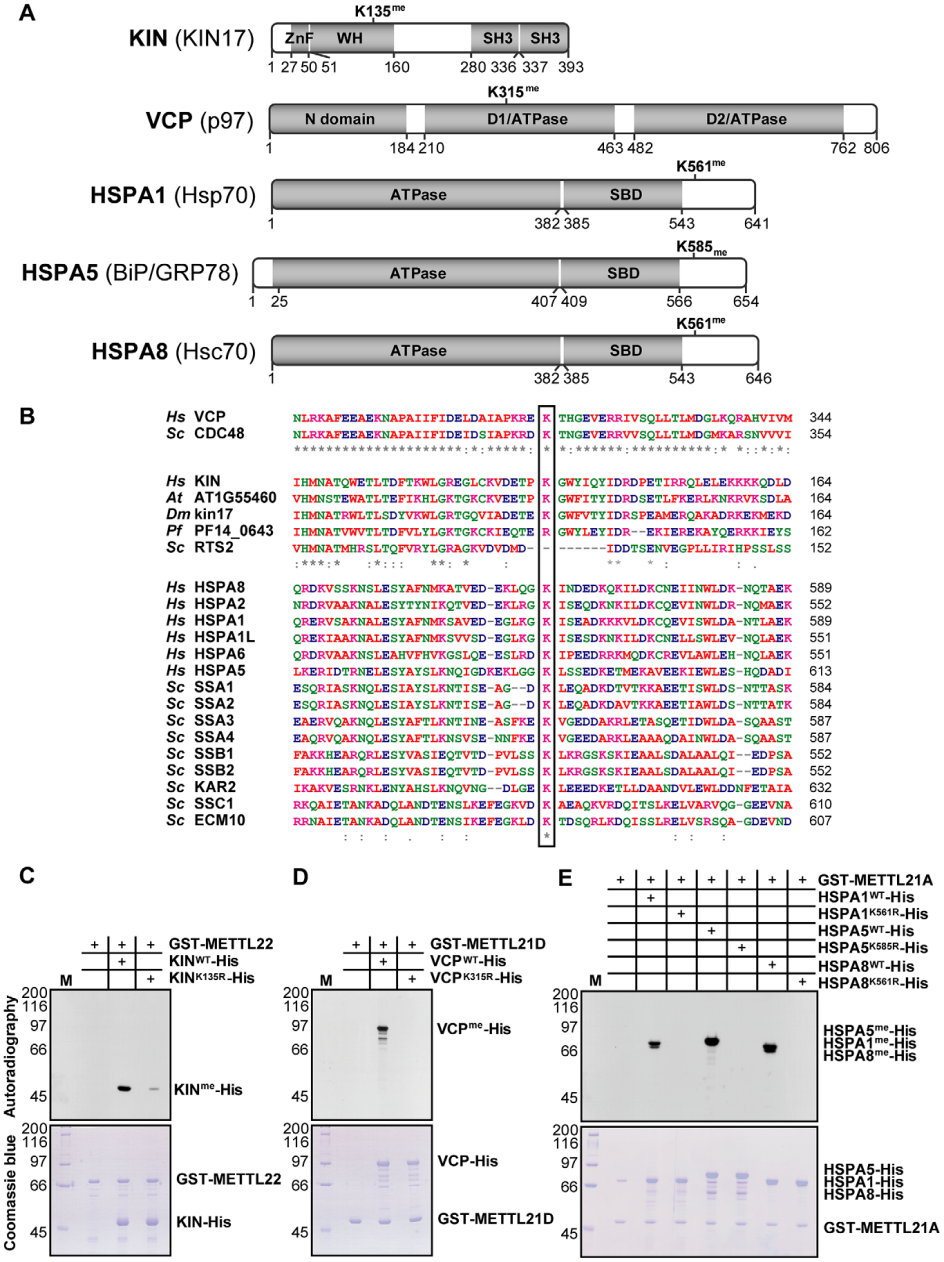
KIN, VCP, and a number of hsp70 isoforms are each trimethylated on lysine residues by specific methyltransferases within this family. (A) Linear representation of all identified substrates with domain architecture. Residues delineating each domain are marked below. ZnF, Zinc Finger; WH, Winged Helix; SH3, Src Homology 3; SBD, substrate binding domain. Position of the methylated lysines is shown above. (B) Multiple sequence alignment of the region surrounding trimethylated lysines (boxed) in human VCP, KIN, and Hsp70 isoforms compared to paralogous genes in various organisms. Hs, *Homo sapiens*; Sc, *Saccharomyces cerevisiae*; Dm, *Drosophila melanogaster*; At, *Arabidopsis thaliana*; Pf, *Plasmodium falciparum*. Color code is as in Figure 1. Strong and weak residue similarity are represented by a colon (:) and period (.), respectively, and asterisk (*) denotes identity. (C–E) In vitro methylation assays with tritium-labeled S-adenosylmethionine of KIN-His with GST-METTL22 (C), VCP-His with GST-METTL21D (D), and three His-tagged hsp70 isoforms (HSPA1, HSPA5, and HSPA8) with GST-METTL21A (E). In each case, substitution of the methylated lysine by an arginine leads to loss of methylation signal as detected by autoradiography. Coomassie staining of the gel shows total proteins loaded onto the gel and serves as control.

Protein database searches were repeated allowing for mono-, di-, and trimethylation of lysine residues (as a variable modification). Of note, lysine trimethylation and acetylation are sometimes mis-annotated due to the closeness in mass of these modifications (+42.0468 Dalton and +42.0105 Dalton, respectively) [43]. Fortunately, the high mass accuracy obtained with the LTQ Orbitrap mass spectrometer was sufficient to distinguish between these PTMs. The most promising hits were a trimethylated lysine on KIN at position 135 in the METTL22 purification, another trimethylated lysine at position 315 on VCP in the METTL21D purification, and a number of trimethylated lysines on multiple Hsp70 isoforms, which correspond to a homologous site, in the METTL21A purification (Figure 3A; see corresponding mass spectra in Figure S3). These methylation sites were highly conserved through evolution (Figure 3B). Conservation of the VCP methylation site K315 is not surprising considering the relative immutability of the overall protein (roughly 70% sequence identity from *Homo sapiens* to *Saccharomyces cerevisiae*). The target lysine 561 in HSPA8/Hsc70 was likewise conserved through evolution and orthologs are found in species as distant as *S. cerevisiae*. Moreover, and as mentioned previously, this site is also retained in a number human Hsp70 paralogs (HSPA1, HSPA1L, HSPA2, HSPA5, and HSPA6). In fact the only conserved residue in this region of loose homology is the target lysine, pointing to a possible important regulatory role for this modification. The target lysine in KIN, K135, is present in a number of species, including *Arabidopsis thaliana* and *Drosophila melanogaster*, but is absent in *Saccharomyces cerevisiae* and *Plasmodium falciparum*. Interestingly, METTL22 orthologs are concurrently absent in species where the corresponding lysine in KIN is not conserved, which further suggests a strong link between the two.

We then proceeded with in vitro methylation assays to confirm the identity of these methylation targets (Figure 3C–3E). A positive signal was observed for each reaction, confirming that these are in fact protein methyltransferases. Moreover, substitution of each identified lysine to an arginine, a relatively conserved substitution, led to the abrogation of the methylation signal. In the case of KIN, the K135R substitution greatly diminished the methylation signal, but did not completely abolish it as with other mutants tested. This could mean that there might be a second methylation site on KIN, but given that no other methylated peptide was ever observed by mass spectrometry, either in the original purification of METTL22 or in the in vitro methylation reaction itself (see corresponding mass spectra in Figure S4), we believe that the residual methylation is more likely to occur on a cryptic site, i.e., one that is not normally methylated in wild-type KIN. Of note, methylation by METTL21A was assayed on three Hsp70 homologs (HSPA1, HSPA5, and HSPA8), but it stands to reason that the modification would also apply to other isoforms where the lysine residue is conserved.

**Figure 4:**
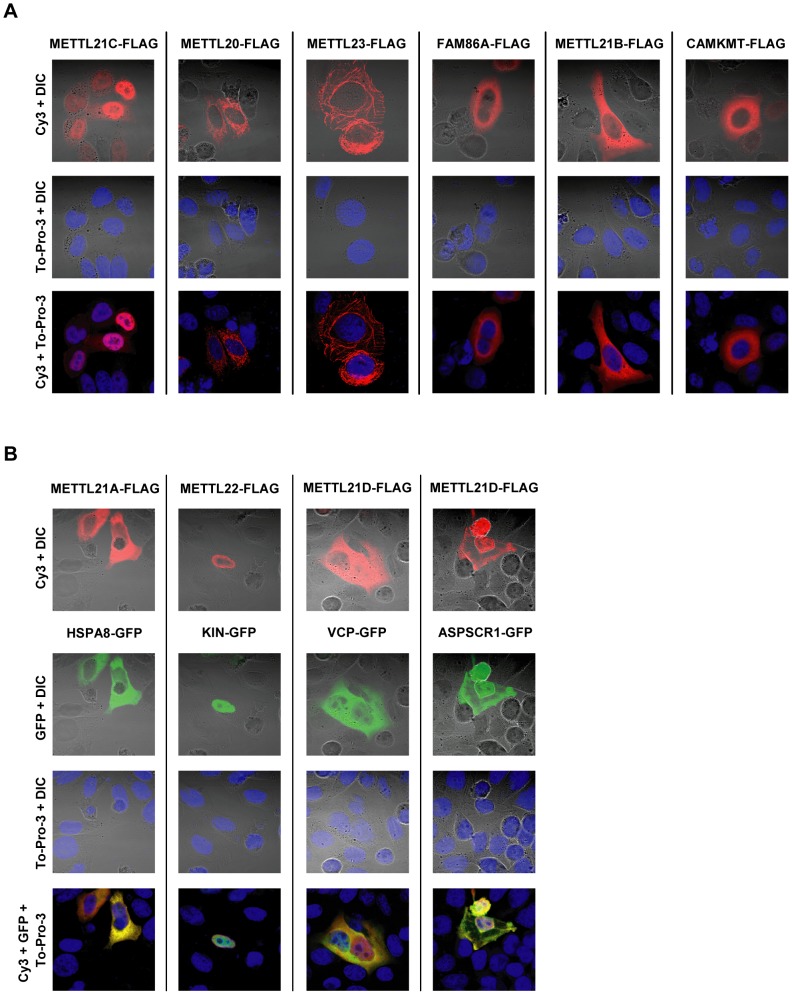
Intracellular distribution of putative methyltransferases and colocalization with identified substrates as shown by immunofluorescence. (A) HeLa cells were transiently transfected with vectors encoding C-terminally FLAG-tagged putative methyltransferases. Localization of the recombinant proteins is revealed by immunofluorescence with a Cy3-labelled antibody. DNA is stained with To-Pro-3 to determine the position of the nucleus, and overall morphology of the cell is shown by Differential Interference Contrast microscopy (DIC). (B) Comparative localization of methyltransferase with associated proteins is shown by transient cotransfection of vectors for the expression of FLAG-tagged methyltransferase and C-terminally GFP-tagged interactors.

To further characterize the function of the methyltransferases, intracellular localization was determined by immunofluorescence. To this end, recombinant FLAG-tagged proteins were expressed in HeLa cells (Figure 4). For most methyltransferases, a marked preference for the cytoplasm was observed, although this trend is reversed in METTL21C and METT22, where the localization is predominantly nuclear. This nuclear distribution could hint at a nucleosomal methylation activity, since it is frequent with most other protein methyltransferases, but none of the four major histones appear to be methylated in vitro by the members of this family (see Figure S5). Localization of METTL18 was never determined, since no significant expression of the recombinant methyltransferase has ever been observed. It is tempting to speculate that this effect could be the result of impaired translation, since METTL18 interacts with, and probably methylates, ribosomal subunit RPL3. Whereas most methyltransferases display a diffuse distribution, METTL20 is concentrated in cytoplasmic granular foci and METTL23 displays internal membrane-like structures.

In the three instances where a methylation target has been identified, the substrate proteins (or associated protein ASPSCR1, in the case of METTL21D) bearing a GFP marker were co-expressed with the corresponding methyltransferase (Figure 4B). All methyltransferases and methyl acceptors more or less colocalize within the same cell compartments. In the case of METTL21A with HSPA8 and METTL21D with VCP or ASPSCR1, the colocalization is nearly perfect. METTL22 and KIN are both present in the nucleus, although METTL22 is clearly more concentrated in the periphery than KIN.

### ASPSCR1/UBXD9 interacts with METTL22 to stimulate methylation of VCP

**Figure 5:**
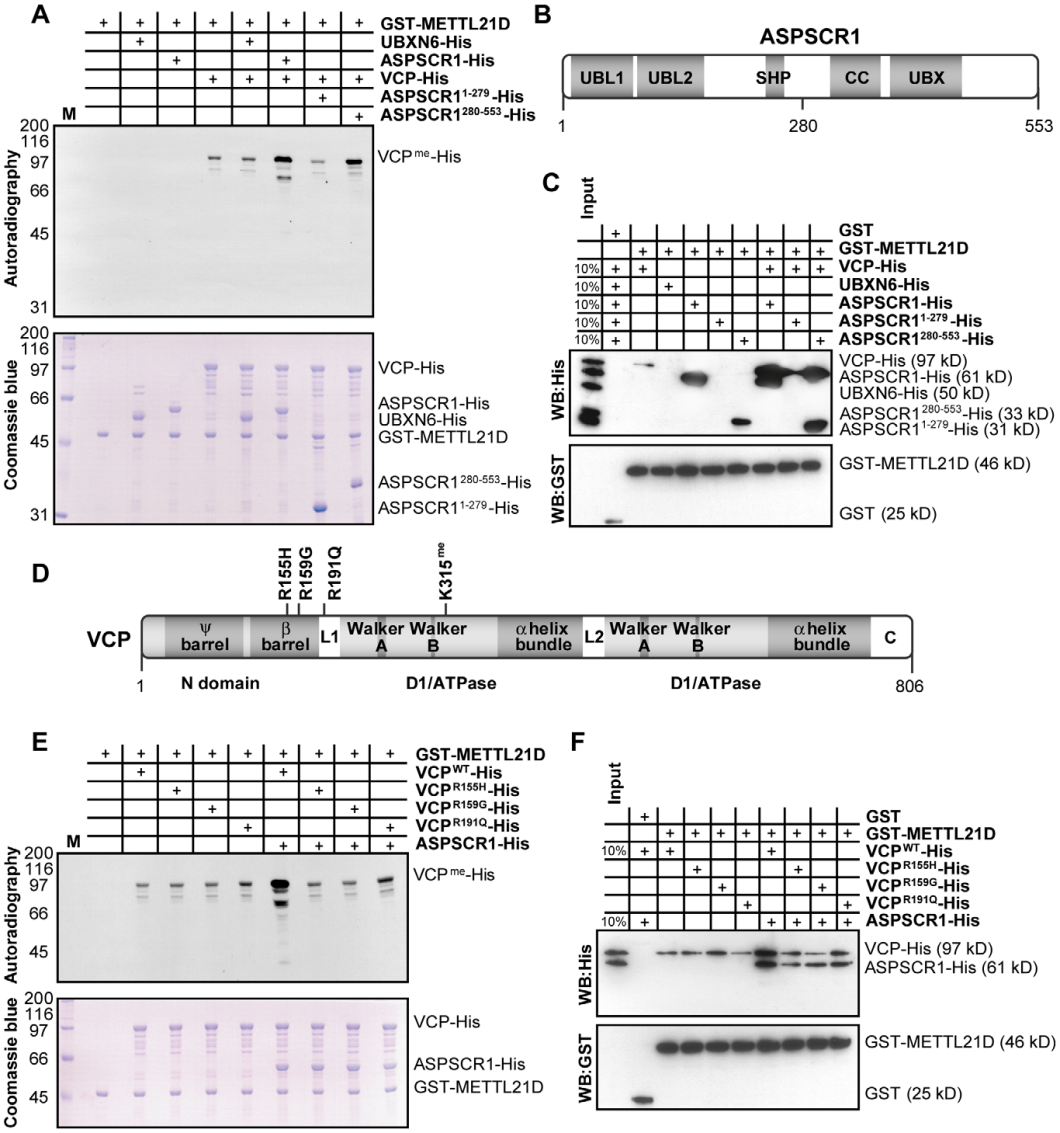
ASPSCR1 promotes methylation of VCP by METTL21D. (A) In vitro methylation assays of VCP-His, UBXN6-His, ASPSCR1-His and fragments of ASPSCR1 with GST-METTL21D. Various combinations of the UBX proteins were added to reactions containing VCP. Coomassie staining of the gel is shown. (B) Linear representation of ASPSCR1 showing domain architecture of the protein (UBL, UBiquitin-Like domain; SHP, SHP box; UBX, UBiquitin regulatory X domain; CC, Coiled-Coil domain) and localization of residue 280 which marks the boundary between the N- and C- terminal fragments used in these experiments. (C) In vitro GST pull-down assays of VCP-His, UBXN6-His, ASPSCR1-His and fragments of ASPSCR1 with GST-METTL21D. Combinations of full-length ASPSCR1 and its fragments were once again employed with VCP. (D) Linear representation of VCP showing domain architecture of the protein (including double Ψ barrel superfold and 4-stranded β barrel of the N-terminal domain, Walker A and B motifs, as well as 4 α helices bundle of ATPase domains D1 and D2 as well as linker regions L1 and L2 and C-terminal domain) and localization of the mutants used in this study as well as the site of methylation. (E) In vitro methylation assays of wild-type VCP-His and IBMPFD and ALS-causing mutations R155H, R159G and R191Q in presence or absence of ASPSCR1-His. (F) In vitro GST pull-down assays of the same combination of proteins as in (E).

Methyltransferases often require cofactors to aid in the modification of their substrates. A good example of this is methylation of spliceosomal Sm proteins by the PRMT5/WD45 complex with the help of pICIn [44]. In the purification of METTL21D, we were able to identify two poorly documented VCP binding proteins, UBXN6/UBXD1 and ASPSCR1/UBXD9. This came as a surprise since VCP was shown to interact with an impressive number of cofactors including the entirety of the UBX (ubiquitin regulatory X) family [45]. Given this apparent specificity, we tested whether these proteins could act as cofactors in the methylation of VCP. As shown in Figure 5A, methylation experiments revealed that neither ASPSRC1 nor UBXN6 could be methylated directly by METTL21D but that only the addition of ASPSCR1, not UBXN6, could enhance methylation of VCP. N- and C-terminal fragments of ASPSCR1 were generated in an effort to determine which domain of the cofactor is responsible for this effect (Figure 5B). To our surprise, we observed that only the C-terminal fragment (residues 280–553), which was previously shown to interact weakly with VCP [46], could enhance VCP methylation in a similar manner as full-length ASPSCR1. An in vitro GST pull-down experiment (Figure 5C) confirms direct binding of the methyltransferase METTL21D to its substrate VCP, but also shows interaction with ASPSCR1, more specifically, to its C-terminal fragment. Furthermore, addition of VCP and ASPSCR1 or VCP and the C-terminal fragment of ASPSCR1 together appear to have a synergetic effect on binding to METTL21D, which could account for the concomitant increase in methylation signal.

Numerous mutations in the VCP gene have been linked with genetic disorders such as Inclusion Body Myopathy with Paget's disease of bone and Fronto-temporal Dementia (IBMPFD) and familial Amyotrophic Lateral Sclerosis (ALS) [47], [48]. Substitutions R155H and R191Q have been implicated in both IBMPFD and ALS. Furthermore, R159G was observed in patient with ALS, although other substitutions targeting arginine 159 were found in patients with IBMPFD (Figure 5D). In vitro methylation assay using recombinant VCP bearing these substitutions was done in order to test whether disease-causing mutations can also impact VCP methylation (Figure 5E). Although all mutant proteins appears to be methylated to a similar degree as wild-type VCP in vitro, the addition of the UBX protein no longer seems to enhance the methylation signal. These results can be explained by the notion the mutants used in this assay, as with most described VCP mutations, reside within the N-terminal domain believed to be involved in cofactor association [49]–[51]. In vitro GST pull-down experiment (Figure 5F) confirms that mutation of VCP has no impact on affinity of METTL21D for its substrate. However, when ASPSCR1 is added to the mix, the synergetic increase in binding is only observed with wild-type VCP.

### Methylation of VCP negatively impacts on its ATPase activity

**Figure 6:**
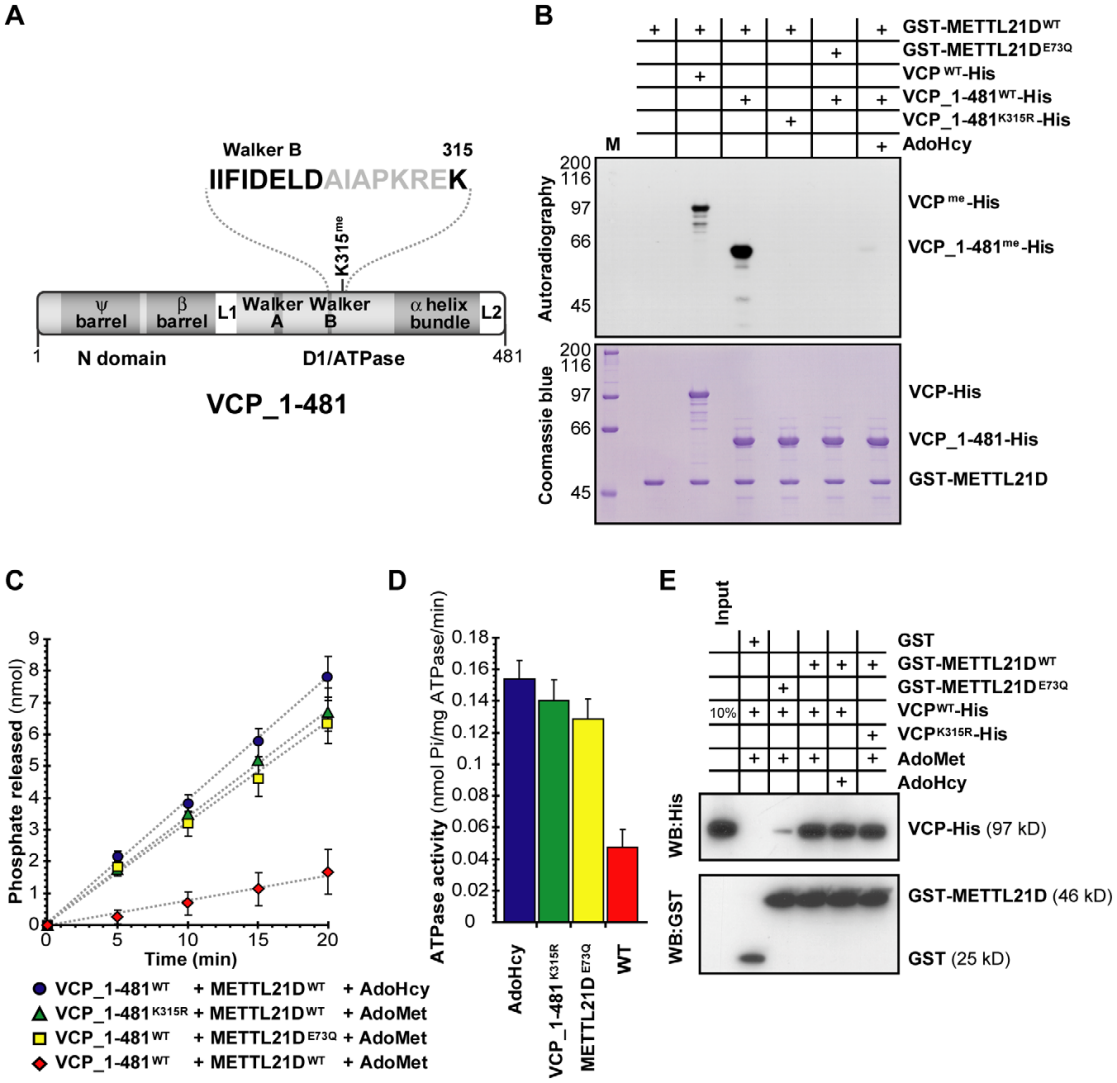
Methylation of VCP decreases the activity of its N-terminal ATPase domain. (A) Linear representation of a fragment of VCP encompassing its N-terminal and first ATPase domain employed in the ATPase assay. Proximity of the methylated lysine to the Walker B motif is highlighted above. (B) In vitro methylation assays of 1–481_VCP-His fragment by GST-METTL21D as compared to full length VCP. (C, D) Colorimetric assays to measure released phosphate (C) and relative ATPase activity (D) by the 1–481 fragment of VCP. The experiment was done in triplicate. Data from the last 3 time points (9 measurements in total for each condition) was compiled to generate the graph shown in (D). (E) In vitro GST pull-down assay of VCP-His with GST-METTL21D. In all experiments the effect of un methylatable VCP mutant K135R, catalytically inactive METTL21D mutant E73Q, and methylation inhibitor S-adenosylhomocysteine is shown.

Given VCP's involvement in disease, we decided to further scrutinize the functional implications of its methylation. This member of the AAA (ATPases Associated with various cellular Activities) family of ATPases contains dual ATPase domains. The methylation site falls in close proximity to the Walker B motif of VCP's first ATPase domain (Figure 6A). Knowing that Walker B motifs are usually involved in ATP hydrolysis, we hypothesized that trimethylation of lysine 315 might affect the ATPase activity of this domain. To test this idea, in vitro ATPase assays were performed with a fragment of VCP spanning its N-terminal and first ATPase domain. The reasoning behind this was that since most of VCP's ATPase activity stems from its second ATPase domain [52], if methylation of K315 only affects the activity of the first ATPase domain, we might not have detected a change in the overall activity of the full-length protein. Before going forward with in vitro ATPase assays, we first verified that the fragment could still be methylated and that methylation could be inhibited by S-adenosylhomocysteine (AdoHcy), a byproduct of methylation that also acts as a methylation inhibitor for most methyltransferases. A catalytically inactive mutated form of the methyltransferase was also created that targets the conserved acidic residue in METTL21D's AdoMet-binding motif (E73Q, see Figure 1C). The results show that the fragment is methylated as efficiently as full-length VCP (Figure 6B). Furthermore, substitution of the VCP fragment by an unmethylatable mutant (K315R), substitution of the methyltransferase by the catalytically inactive mutant (E73Q), or even addition of AdoHcy all resulted in nearly complete inhibition of methylation.

We then performed the in vitro ATPase assay and found that when a wild-type VCP fragment is pre-incubated with wild type METTL21D and the methyl donor, AdoMet, a significant decrease in ATPase activity was detected as compared to three separate control reactions where either the methyltransferase is replaced by its E73Q mutant; VCP is replaced with its unmethylatable mutant (K315R); or the methyl donor is replaced with AdoHcy (Figure 6C and 6D).

A possible interpretation of this finding is that methylation of VCP does not inhibit the ATPase activity *per se*, but that binding of the methyltransferase itself hinders the ATPase function. To eliminate this possibility, in vitro GST pulldowns were carried out (Figure 6E). Although we did observe a decreased binding between methyltransferase and substrate when METTL21D is replaced by the E73Q mutant, addition of AdoHcy and mutation of VCP lysine 315 do not appear to affect the interaction when compared to wild-type METTL21D and wild-type VCP in presence of AdoMet. This result confirms the conclusion that methylation of VCP directly modulates the ATPase activity of its first ATPase domain. Additionally, these experiments were repeated with a full-length form of VCP whose second ATPase domain has been inactivated by a mutation targeting a critical residue within the Walker B motif (E578Q; Figure S6) [53]. Again, a decrease in ATPase activity is observed when VCP_E578Q is preincubated with METTL21D and AdoMet.

## Discussion

The data presented here bring an entirely new group of protein methyltransferases into light and suggest a role for this post-translational modification in modulating chaperone function. Hsp70 isoforms have been known to be methylated both on arginine and lysine residues for quite some time [54], [55], but up until now the exact sites of these modifications and the enzymes responsible for them had remained elusive. The role of these modifications is also uncertain, but we speculate that they may help direct specificity of these chaperones towards substrates and cofactors. Evidence for this could be derived from the AP-MS data presented here. Indeed, METTL21A, the only known Hsp70 methyltransferase identified so far, copurified with a number of Hsp70 isoforms but few cofactors aside from Hsp110s. The closely related METTL21B also copurified with significant amounts of Hsp70 but this time appeared to be complexed with STIP1/Hsp90 or CCT chaperonin. That differential methylation by these enzymes drives Hsp70 specificity is a hypothesis that remains to be tested.

What is certain based on the results presented in this article is that the ATPase activity of another seemingly unrelated chaperone, VCP, can be modulated by METTL21D-dependent lysine trimethylation. As with Hsp70s, VCP has also been shown to be extensively modified, mostly by phosphorylation and acetylation [56]–[60]. In this report, we demonstrate that methylation of the VCP requires a novel, specific methyltransferase, which in turn seems to be highly conserved throughout evolution. Indeed, tandem-affinity purification of a yeast homolog of METTL21D, Nnt1p, led to the identification of the yeast homolog of VCP, Cdc48p (Figure S7 and Table S2), hinting at the importance of this interaction.

Strickingly, methylation of VCP is further enhanced by direct interaction of the methyltransferase with ASPSCR1, a poorly characterized VCP cofactor, and this effect appears to require the C-terminal half of ASPSCR1. Mutations in the VCP gene have been linked to autosomal dominant disorders Inclusion Body Myopathy with Paget's disease of bone and Fronto-temporal Dementia (IBMPFD) and familial Amyotrophic Lateral Sclerosis (ALS) [47], [48]. Most VCP mutations reside within the N-terminal domain, which has been proposed to be involved in cofactor association [49]–[51].

Substitutions R155H, R159G and R191Q have no impact on the in vitro methylation of the protein. However, addition of ASPSCR1 no longer appears to increase the methylation of mutant VCP as compared to the wild-type protein. This observation opens up a whole new area of investigation in understanding the molecular physiopathology of IBMPFD and familial ALS. It may therefore be of interest to assess the relative methylation of VCP in affected patients as compared to healthy individuals. Many studies were performed to define how these disease mutations affect the function of VCP. From a biochemical point of view, the most promising alteration concerned the increased ATPase activity that may reflect structural changes induced by ATP binding [61], [62]. Methylation of VCP by METTL21D is shown here to significantly decrease activity of the first ATPase domain of this chaperone. This modification could eventually help modulate enzymatic activity of VCP that has gone haywire due to mutation.

Our work on the KIN protein, which eventually led to the discovery of its associated methyltransferase METTL22, began when it was detected in the interactome of a number of prefoldins (see supporting data in [32]). Thus, even though KIN is not known to have chaperone activity, it still appears to interact with chaperones and potentially affect their activity. The exact function of KIN is still a matter of debate. This DNA and RNA binding protein has been assigned a role in DNA repair and/or replication [63]–[66] and possibly mRNA processing as suggested by its identification in a number of proteomic analyses of the spliceosome [67], [68]. The role of the herein identified methylation will likely go hand in hand with the function of the winged helix domain in which it resides. Interestingly, yet another winged helix-containing protein was detected in the METTL22 purification, FOXK1. In this case, the function of the winged helix is known since it is required for DNA binding of this transcription factor. If METTL22 is shown to methylate FOXK1 as it did with KIN, this may in turn point to a more complex regulation of winged helix factors.

Advances in proteomics have helped to catalog various post-translational modifications of the proteome, and it now seems evident that chaperones contain several occurrences of such modifications. Recent identification of Hsp90 methylation by lysine methyltransferase SMYD2 is further evidence of the significance of this modification in regulating chaperone function [69]. Just like post-translational modifications of histone tails were shown to modulate binding to multiple chromatin remodeling, transcription, and mRNA processing factors, we believe that chaperone modifications may compose a similar code to help define specificity of discrete subsets to their seemingly innumerable effectors. Further decrypting this “chaperone code” is now required to understand how the functional organization of the proteome is orchestrated.

## Materials and Methods

### Generation of cell lines for expressing TAP-tagged polypeptides

Coding sequences for methyltransferases discussed in this article were obtained from the I.M.A.G.E. consortium clone library (Thermo Scientific). The corresponding cDNAs were cloned into the mammalian expression vector pMZI [70] carrying a TAP tag at its C-terminus [71], [72]. Stable human embryonic kidney cell lines (EcR-293; derived from HEK293) carrying these constructs were produced as described previously [28], [33].

### Tandem affinity purification

Induction for 48 hours with 3 µM ponasterone A (Life Technologies) was used to express the TAP-tagged proteins. Whole cell extracts prepared from induced and non-induced stable EcR-293 cell lines were subjected to purification by the TAP procedure as described previously [28], [33].

### Protein identification by mass spectrometry

TAP eluates were desalted and concentrated on Amicon Ultra 10K centrifugal filter units (Millipore) and resolved on NuPAGE 4–12% Bis-Tris Gel (Life Technologies). The gels were silver-stained, and the entirety of the tracks where the eluates had migrated were cut in about 20 slices that were subsequently digested with trypsin as described previously [28], [33]. The resulting tryptic peptides were purified and identified by LC-tandem mass spectrometry (MS/MS) using a microcapillary reversed-phase high pressure liquid chromatography coupled to a LTQ Orbitrap (ThermoElectron) mass spectrometer with a nanospray interface. The peak list files were generated with extract_msn.exe (version February 15, 2005) using the following parameters: minimum mass set to 600 Da, maximum mass set to 6000 Da, no grouping of MS/MS spectra, precursor charge set to auto, and minimum number of fragment ions set to 10. Protein database searching was performed with Mascot 2.3 (Matrix Science) against the human NCBInr protein database (version April 2, 2009). There are 10,427,007 sequences in this database. The mass tolerances for precursor and fragment ions were set to 10 ppm and 0.6 Da, respectively. Trypsin was used as the enzyme allowing for up to two missed cleavages. Carbamidomethyl, oxidation of methionine, mono-, di-, and trimethylation of lysine were allowed as variable modifications. In cases where multiple gene products were identified from the same peptide set, all were unambiguously removed from the data set. In the case of multiple isoforms stemming from a unique gene, the isoform with the best sequence coverage was reported. Proteins identified on the basis of a single peptide were also discarded.

Reliability assessment of protein-protein interactions was performed by our previously published software Decontaminator [73]. A set of 17 pairs of matched non-induced bait expression (control) and induced bait expression vectors was used for the algorithm training. Those were chosen on the basis of the absence of leaky expression of the tagged protein in the non-induced experiments. Decontaminator builds Bayesian probabilistic models of the Mascot scores [74] for each protein observed in the training set. It then assigns a p-value to each bait-prey interaction by computing the significance of the observed prey Mascot score compared to its corresponding control Mascot score model. A False Discovery Rate (FDR) is then calculated for each protein-protein interactions in the dataset using a leave-one-out scheme. All interactions with a FDR below 10% were reported as bait-specific, resulting in a dataset of 234 interactions. In other words, less than 24 interactions are expected to be the consequence of contamination.

### In vitro methylation assay

The protocol was slightly modified from Inamitsu et al. [75]. Coding sequences of methyltransferases were cloned into pGEX-4-T1 vector (GE Healthcare). Coding sequences of putative methylation substrates were cloned into pET-23a(+) vector (EMD Chemicals). All vectors were transformed in One Shot BL21 Star (DE3) (Life Technologies), and protein synthesis was induced with IPTG. Bacteria were harvested by centrifugation, and pellets were lysed with the use of an IEC French Press (Thermo Scientific). The resulting GST- and 6xHis-fusion proteins were purified using Glutathione Sepharose 4B (GE Healthcare) and Ni-NTA Agarose (QIAGEN) beads, respectively, according to the manufacturers' specifications. In each reaction described in this article, 1 µg of GST-tagged methyltransferase was incubated with 2.5 µg His-tagged substrate and 5 µCi of S-[methyl-^3^H]-Adenosyl-L-methionine (81.7 Ci/mmol; PerkinElmer) in 50 µl of PBS for 90 min at 37°C. The samples were resolved on 10% acrylamide gels that were stained with Coomassie to show total amounts of proteins. Gels were then treated with EN^3^HANCE (PerkinElmer) according to the manufacturer's specifications. Tritium-based methylation signals were detected by autoradiography with four hours of exposure on Amersham Hyperfilm MP (GE Healthcare) at −80°C. Alternatively, an assay was produced with unlabeled S-adenosyl-L-methionine that was resolved on NuPAGE 4–12% Bis-Tris Gel (Life Technologies) and Coomassie stained. The bands corresponding to the His-tagged substrates were excised, trypsin digested, and analyzed as described above.

### Immunofluorescence

HeLa S3 (CCL-2.2 ATCC) cells were grown on Lab-Tek II chamber slides (Nalge Nunc) and co-transfected with FLAG and GFP expressing vectors (p3XFLAG-CMV-14 and pGFP2-N1; Sigma-Aldrich & PerkinElmer Life Sciences, respectively) using Lipofectamine 2000 according to the manufacturer's specifications (Life Technologies). Twenty-four hours following transfection, cells were fixed with 3.7% formaldehyde in Phosphate-Buffered Saline (PBS) and permeabilized with 0.3% Triton X-100 PBS. Slides were then fixed in 5% donkey serum PBS for 1 hour, incubated with 1∶200 monoclonal FLAG antibody (M2; Sigma-Aldrich) in 5% donkey serum PBS for 90 min, and then incubated for an additional hour with 1∶50 Cy3-conjugated donkey anti-mouse IgG secondary antibody (Jackson ImmunoResearch) in 5% donkey serum PBS. Slides were washed three times with PBS for 5 min after each step. DNA was stained with TO-PRO-3 (Molecular Probes). Slides were mounted using ProLong Gold antifade reagent (Life Technologies). Images were acquired with an LSM 700 confocal laser scanning microscope at 63× magnification and analyzed using ZEN 2010 software (Zeiss, Toronto, Canada).

### In vitro GST pull-down assay

The assay was based on the protocol described in Zwijsen et al. [76]. Briefly, 500 ng of GST-tagged METTL21D was incubated for 1 hour at 4°C with 100 ng of His-tagged VCP, ASPSCR1 or UBXN6, and 25 µl Glutathione Sepharose 4B beads (GE Healthcare) in 1 ml of binding buffer (50 mM NaCl, 50 mM HEPES-KOH pH 7.6, 0.1% NP-40, 0.5% charcoal-stripped FBS) complemented with complete EDTA-free protease inhibitor cocktail (Roche). The beads were washed three times by centrifugation with the same buffer and the bound proteins were eluted by boiling for 5 min in sample buffer, and separated on NuPAGE 4–12% Bis-Tris Gel (Life Technologies). Binding of His-tagged proteins was detected by Western blot analysis using mouse monoclonal anti-6X His tag-antibody (abcam). A second blot was made with rabbit polyclonal anti-GST antibody (abcam) to ensure that comparable amounts of GST-tagged baits were purified by the pull-down.

### ATPase assay

PiColorLock Gold Phosphate Detection System (Innova Biosciences) was used to quantify ATPase activity in vitro. Three micrograms of a His-tagged fragment corresponding to the first 481 residues of VCP were incubated beforehand with 2 µg of GST-METTL21D and 0.5 mM S-adenosyl-L-methionine for 30 min at 37°C in 100 µl of 0.1 M Tris pH 7.5. All assays were performed in triplicate. Independent experiments were carried out where the VCP fragment was replaced by an unmethylatable mutant (K315R); the methyltransferase was replaced by a catalytically inactive mutant (E73Q); or the methyl donor, S-adenosyl-L-methionine, was replaced by a methylation inhibitor, S-adenosyl-L-homocysteine. The rest of the protocol followed the guidelines provided by the manufacturer.

### Phylogenetic analysis

All available ortholog sequences of PRMTs and of the family of 10 putative methyltransferases in the UniProt database [77] were aligned using the ClustalW2 multiple sequence alignment software (version 2.0.12) [78]. The most conserved region of the alignment was then selected to build an unrooted phylogenetic tree through the Jalview software [79] using the neighbor-joining algorithm [80] with the BLOSUM62 substitution matrix [81]. Orthologs forming monophyletic groups with their respective human sequences were collapsed to a single node in the phylogenetic tree shown in Figure 1A.

## Supporting Information

Figure S1 Jalview visualization of the multiple sequence alignment of all available ortholog sequences of PRMTs and of the family of 10 putative methyltransferases in the UniProt database. The most conserved region of the alignment selected for phylogenetic analysis is delimited in red. Sequence names are in the following format: human ortholog protein name, tr: TrEMBL or sp: SWISS-PROT database, UniProt entry, UniProt entry name.
(PNG)

Figure S2 Phylogenetic tree generated by Jalview of all available ortholog sequences of PRMTs and of the family of 10 putative methyltransferases in the UniProt database. Tree node labels are in the following format: Human ortholog protein name, tr: TrEMBL or sp: SWISS-PROT database, UniProt entry, UniProt entry name.
(PNG)

Figure S3 Annotated spectra for methylated peptides identified from TAP eluates of methyltransferases.
(TIF)

Figure S4 Annotated spectra for methylated peptides identified from in vitro methylation reactions.
(TIF)

Figure S5 In vitro methylation of a mix of histones (H2A, H2B, H3, H4) with every putative methyltransferase discussed in this paper. PRMT6 serves as a positive control.
(TIF)

Figure S6 ATPase assay using a form of VCP bearing a catalytically inactivating mutation in its second ATPase domain. All experiments were done in same conditions as in Figure 6. (A) Linear representation of VCP showing domain architecture of the protein and localization of methylation site as well as E578Q mutation employed to inactivate the second ATPase/D2 domain. (B) In vitro methylation assays of VCP_E578Q-His by GST-METTL21D as compared to wild-type VCP. Colorimetric assays to measure released phosphate (C) and relative ATPase activity (D) of VCP_E578Q. The experiment was done in triplicate. Data from the last 3 time points (9 measurements in total for each condition) was compiled to generate the graph shown in (D). (E) In vitro GST pull-down assay of VCP-His with GST-METTL21D. In all experiments the effect of VCP mutants K135R and K315A, METTL21D mutant E73Q, and S-adenosylhomocysteine is shown.
(TIF)

Figure S7 Purification of TAP-tagged NNT1 from a constitutively expressing yeast strain as compared to untransformed wild-type strain BY4741. Tagged baits and major interactors are marked.
(TIF)

Table S1 Detailed mass spectrometry data and FDR scores for a selection of protein interactions identified in the purification of human TAP-tagged putative methyltransferases.
(XLS)

Table S2 Detailed mass spectrometry data for protein interactions detected in the purification of *Saccharomyces cerevisiae* TAP-tagged NNT1. Positive hits were identified on the basis of a comparison with control strain BY4741.
(XLS)
