# Supplementary figures and images for: A Newly Uncovered Group of Distantly Related Lysine Methyltransferases Preferentially Interact with Molecular Chaperones to Regulate Their Activity
Source: PLoS Genet. 2013 Jan 17;9(1):e1003210. doi: 10.1371/journal.pgen.1003210 (PMC3547847; doi:10.1371/journal.pgen.1003210)

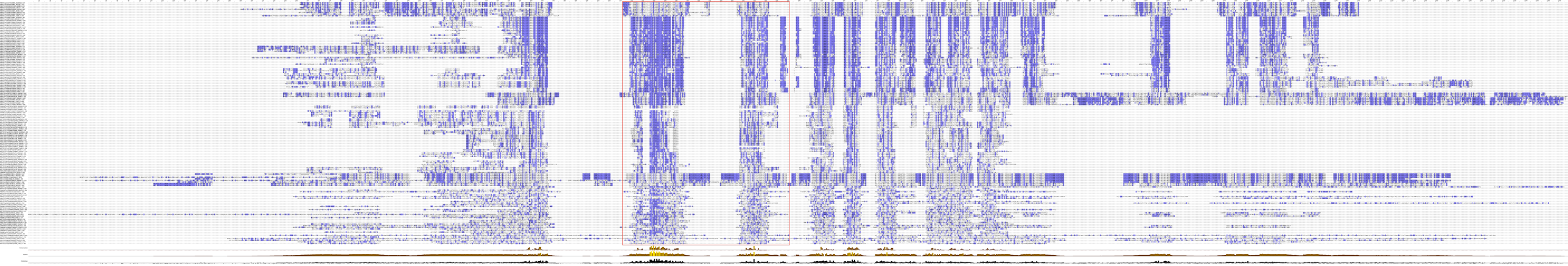

Supplement: Figure S1 — Jalview visualization of the multiple sequence alignment of all available ortholog sequences of PRMTs and of the family of 10 putative methyltransferases in the UniProt database. The most conserved region of the alignment selected for phylogenetic analysis is delimited in red. Sequence names are in the following format: human ortholog protein name, tr: TrEMBL or sp: SWISS-PROT database, UniProt entry, UniProt entry name. (PNG) [file pgen.1003210.s001.png]

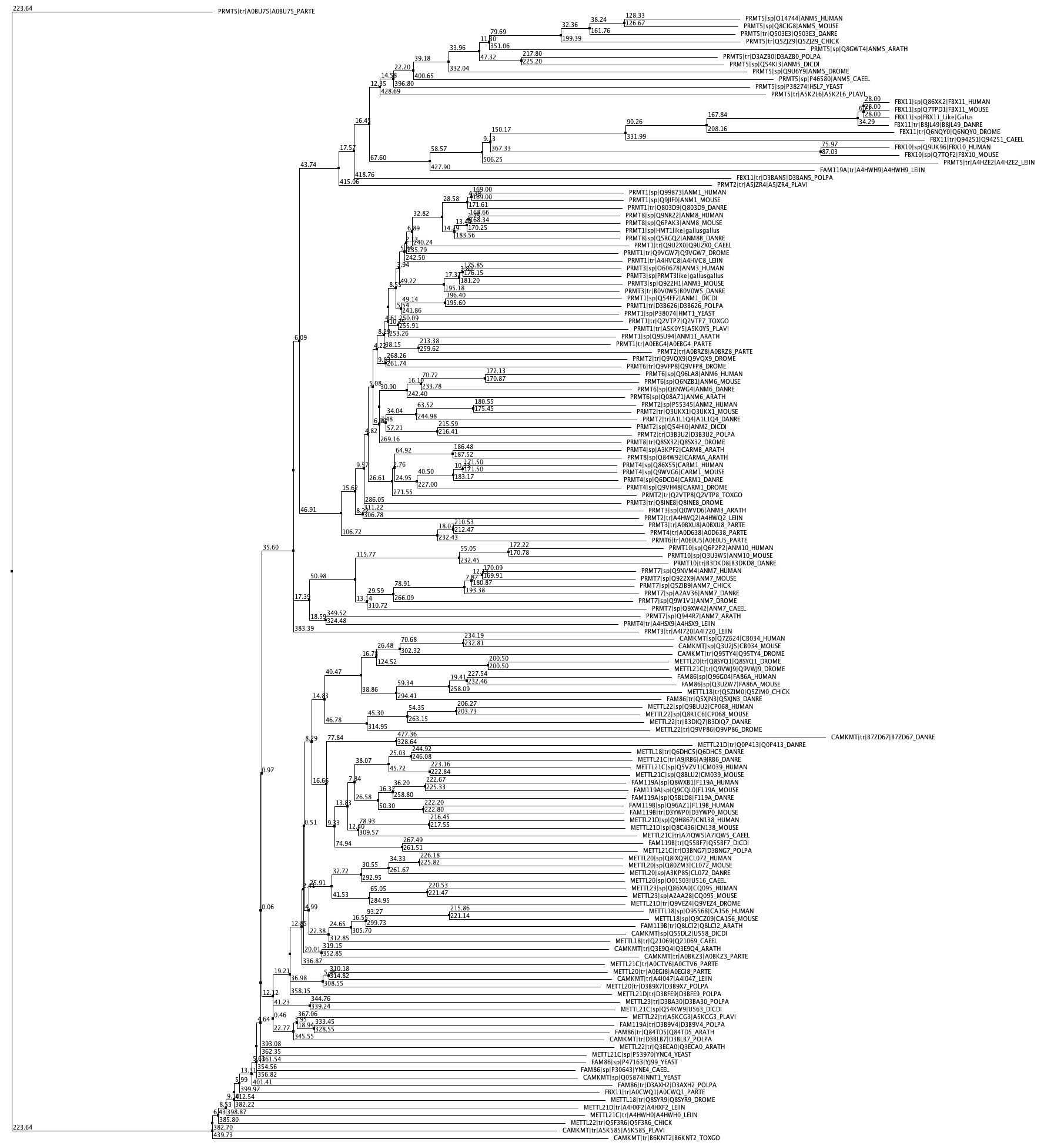

Supplement: Figure S2 — Phylogenetic tree generated by Jalview of all available ortholog sequences of PRMTs and of the family of 10 putative methyltransferases in the UniProt database. Tree node labels are in the following format: Human ortholog protein name, tr: TrEMBL or sp: SWISS-PROT database, UniProt entry, UniProt entry name. (PNG) [file pgen.1003210.s002.png]

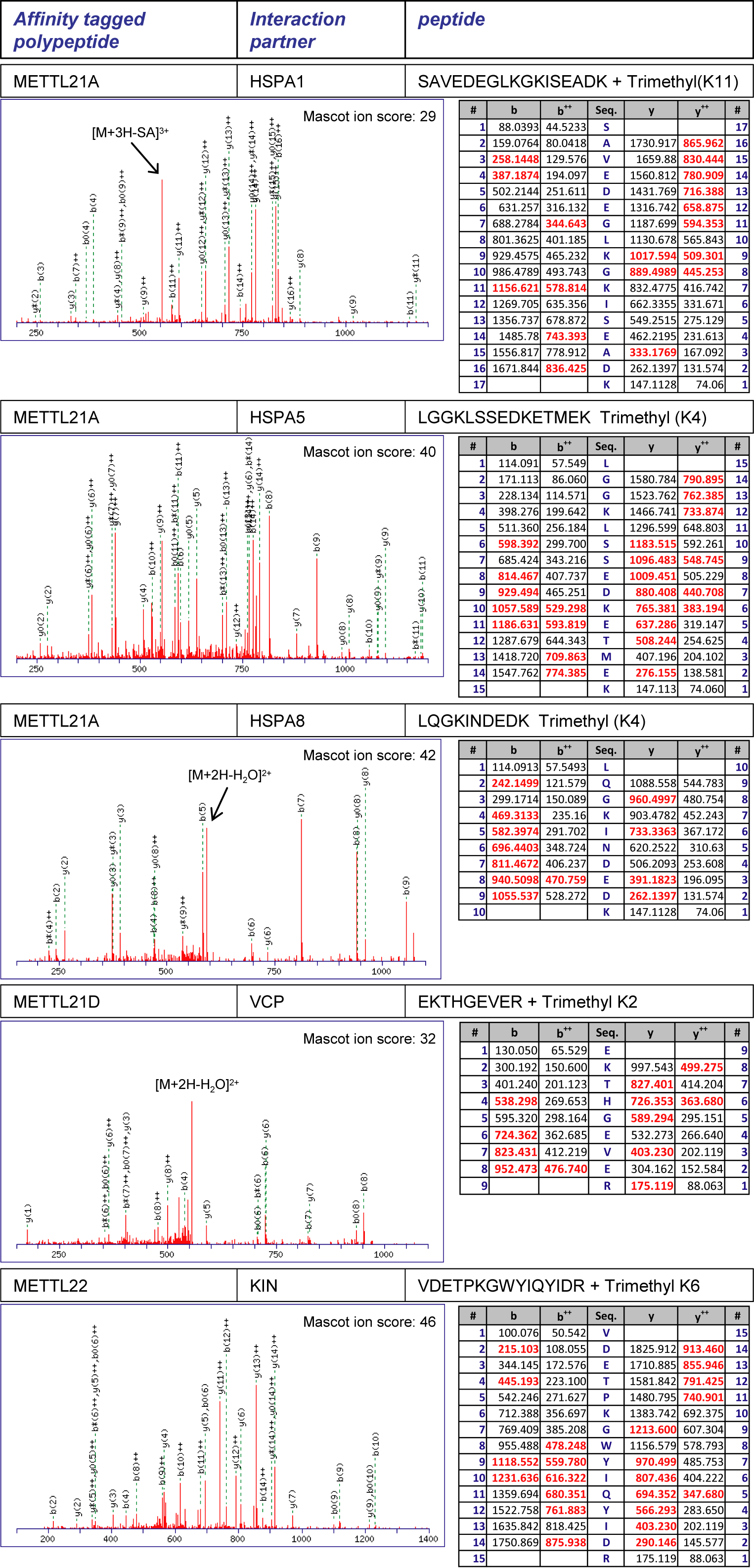

Supplement: Figure S3 — Annotated spectra for methylated peptides identified from TAP eluates of methyltransferases. (TIF) [file pgen.1003210.s003.tif]

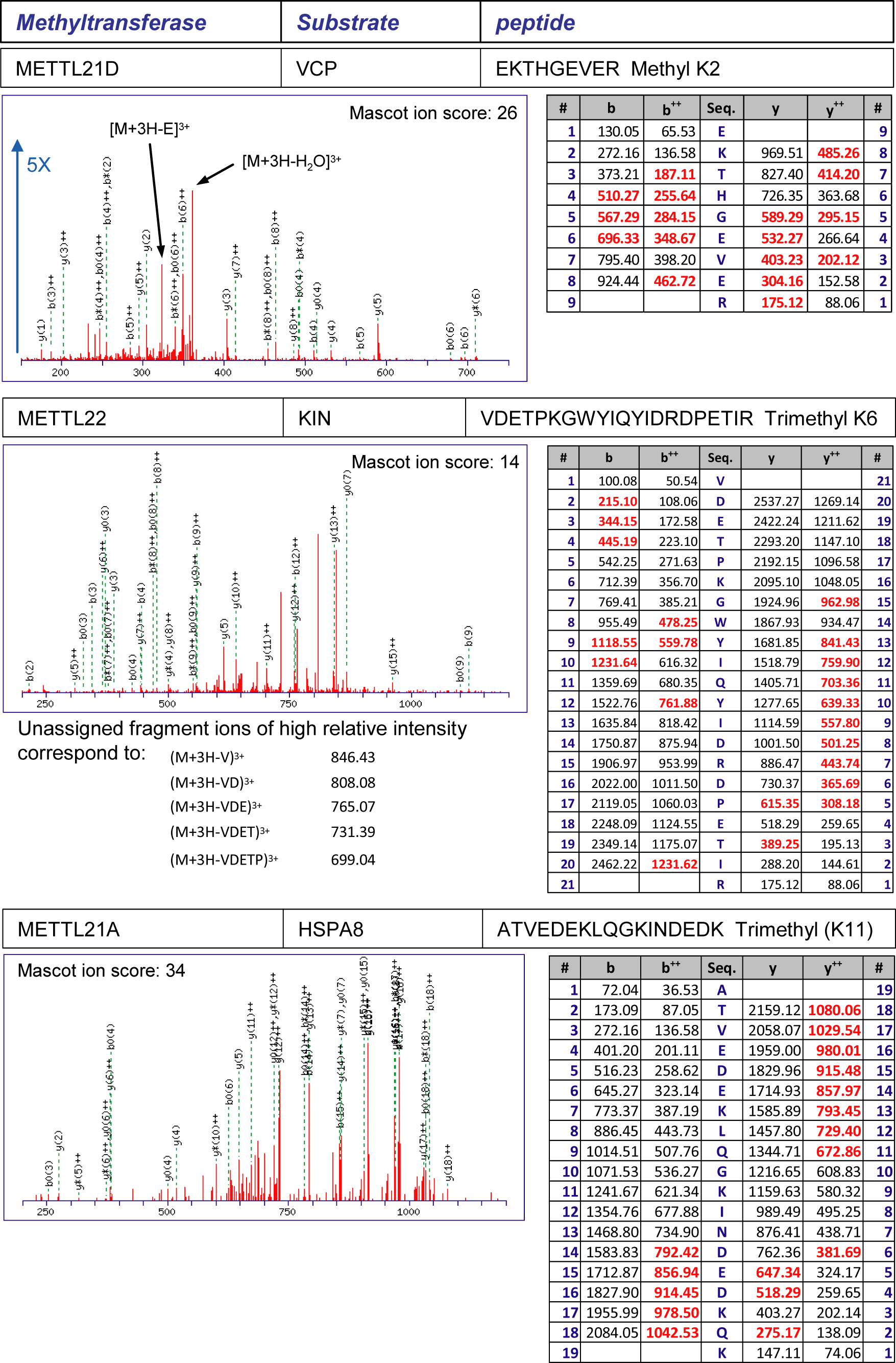

Supplement: Figure S4 — Annotated spectra for methylated peptides identified from in vitro methylation reactions. (TIF) [file pgen.1003210.s004.tif]

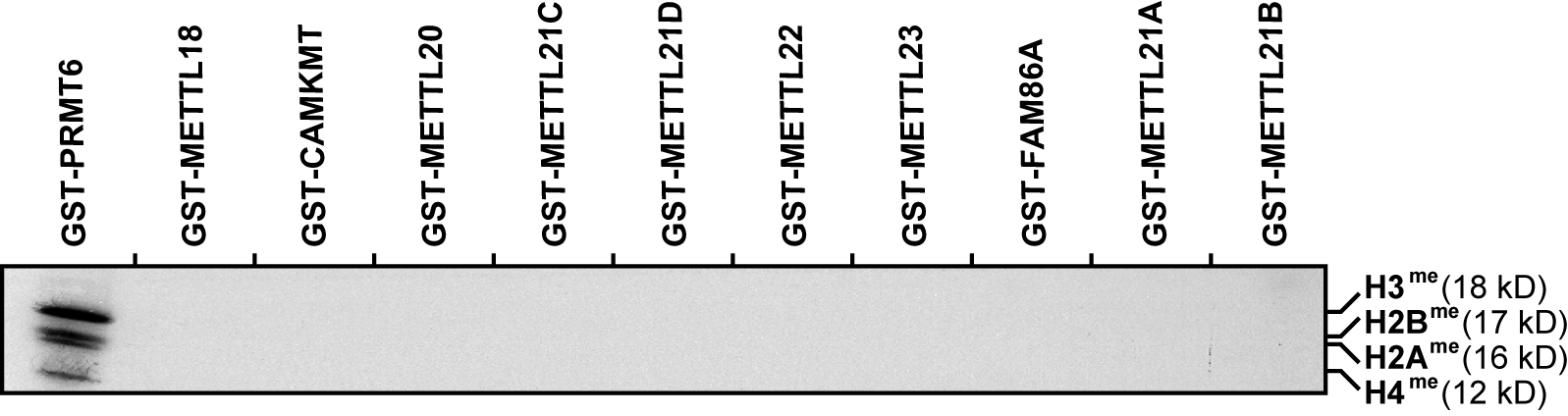

Supplement: Figure S5 — In vitro methylation of a mix of histones (H2A, H2B, H3, H4) with every putative methyltransferase discussed in this paper. PRMT6 serves as a positive control. (TIF) [file pgen.1003210.s005.tif]

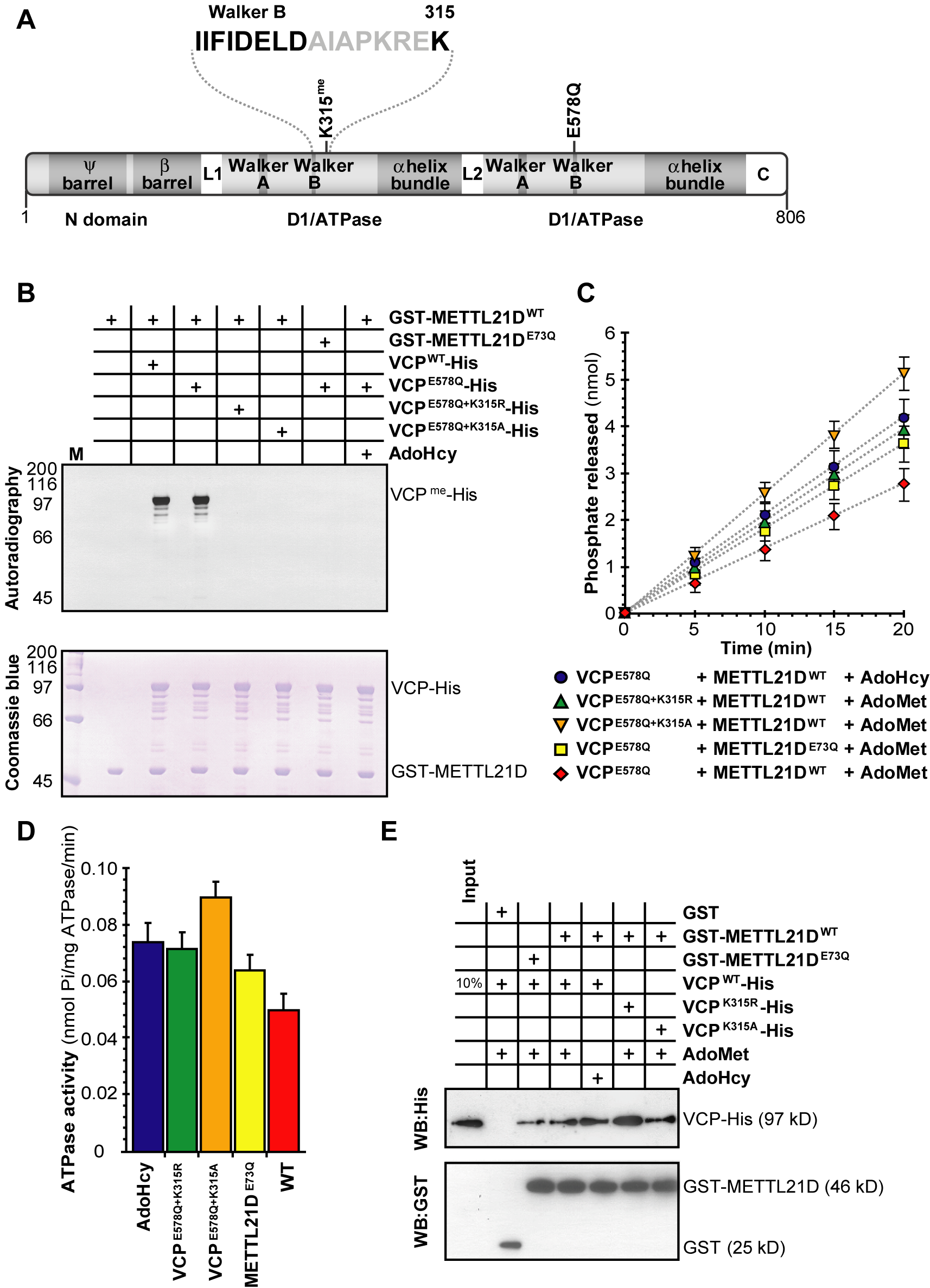

Supplement: Figure S6 — ATPase assay using a form of VCP bearing a catalytically inactivating mutation in its second ATPase domain. All experiments were done in same conditions as in Figure 6. (A) Linear representation of VCP showing domain architecture of the protein and localization of methylation site as well as E578Q mutation employed to inactivate the second ATPase/D2 domain. (B) In vitro methylation assays of VCP_E578Q-His by GST-METTL21D as compared to wild-type VCP. Colorimetric assays to measure released phosphate (C) and relative ATPase activity (D) of VCP_E578Q. The experiment was done in triplicate. Data from the last 3 time points (9 measurements in total for each condition) was compiled to generate the graph shown in (D). (E) In vitro GST pull-down assay of VCP-His with GST-METTL21D. In all experiments the effect of VCP mutants K135R and K315A, METTL21D mutant E73Q, and S-adenosylhomocysteine is shown. (TIF) [file pgen.1003210.s006.tif]

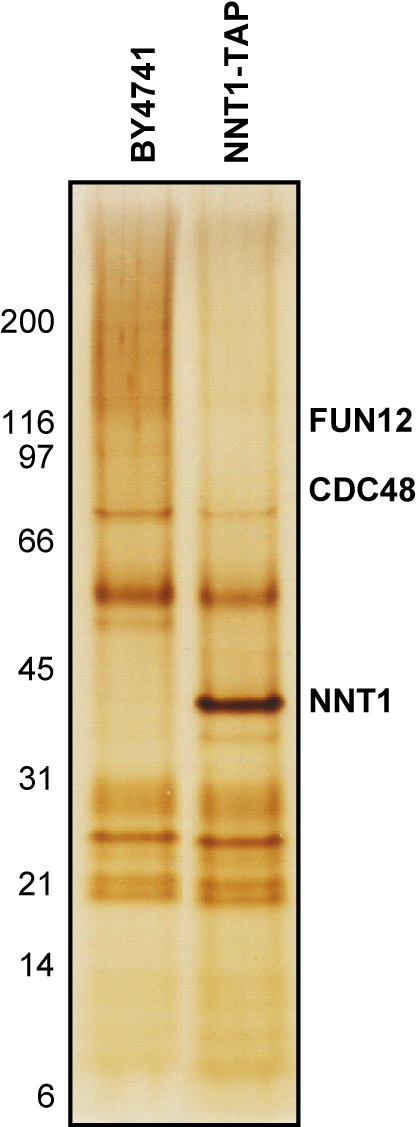

Supplement: Figure S7 — Purification of TAP-tagged NNT1 from a constitutively expressing yeast strain as compared to untransformed wild-type strain BY4741. Tagged baits and major interactors are marked. (TIF) [file pgen.1003210.s007.tif]
